# DPP-4 Inhibitors in Female Cancers: Opportunities for Drug Repurposing

**DOI:** 10.3390/cimb48050445

**Published:** 2026-04-24

**Authors:** Hiba F. Muddather, Zsuzsanna Schelz, István Zupkó

**Affiliations:** 1 Institute of Pharmacodynamics and Biopharmacy, Faculty of Pharmacy, University of Szeged, Eötvös u. 6, 6720 Szeged, Hungary; hiba.161991@hotmail.com (H.F.M.); schelz.zsuzsanna@szte.hu (Z.S.); 2 Department of Clinical Pharmacy and Pharmacy Practice, Faculty of Pharmacy, University of Gezira, Hospital Street, Wad Madani 21112, Sudan

**Keywords:** DDP4, female malignancies, antidiabetic, drug repurposing

## Abstract

Female malignancies, including breast, cervical, ovarian, and endometrial cancers, remain a significant health challenge. Meanwhile, treatment options for advanced-stage remain limited. Drug repurposing has emerged as a promising approach to accelerate the development of effective cancer therapies using existing medications. Growing evidence indicates that metabolic disorders such as type 2 diabetes mellitus are linked with an elevated risk of tumors, highlighting antidiabetic drugs as potential anticancer agents. Among these, inhibitors of dipeptidyl peptidase 4 (DPP4) have attracted attention as potential therapeutic candidates, due to their diverse biological functions in glucose metabolism, inflammation, immune regulation, and tumor biology. This review summarizes current epidemiological, preclinical, and clinical evidence regarding the role of DPP4 in female cancers and the therapeutic potential of DPP4 inhibitors. Studies demonstrate that DPP4 influences key oncogenic processes, including proliferation, invasion, metastasis, immune modulation, and metabolic reprogramming. However, available data on DPP4 inhibition and its influence in cancer therapy are controversial and scarce. Further mechanistic studies and well-designed clinical investigations are required to clarify their safety and clinical applicability in the management of female malignancies.

## 1. Introduction

Female malignancies, encompassing breast, cervical, ovarian, endometrial, and uterine cancers, continue to pose significant health challenges worldwide [1]. Global demographic and socioeconomic changes, particularly population growth and aging, are associated with a steady increase in the burden of these malignancies [2,3]. In 2022, approximately 2.3 million women were diagnosed with breast cancer globally, making it the most prevalent cancer among women. Cervical cancer accounts for the fourth most common cancer in women [4]. While ovarian and uterine cancers occur at a lower frequency, they remain devastating malignancies of the female reproductive system [5]. Survival outcomes also vary substantially across these malignancies. Breast cancer generally has favorable outcomes, with 5-year relative survival rates exceeding 85% in some regions due to improvements in early detection and targeted therapies [6]. In contrast, ovarian cancer continues to have a significantly poorer prognosis, with five-year relative survival rates below 50%, because most cases are diagnosed at advanced stages [7]. Cervical and endometrial showed intermediate survival outcomes that largely depend on the stage at diagnosis and accessibility to treatments [6,8]. Despite advancements in early detection and prevention, treatment options for advanced-stage gynecologic cancers remain limited, emphasizing the need for innovative therapeutic strategies. A summary of epidemiological characteristics and current anticancer recommendations for major female malignancies is presented in Table 1.

Drug repurposing has emerged as a promising approach to address current therapeutic limitations. By identifying new therapeutic uses for existing medications, researchers can expedite the availability of effective treatments, often at a lower cost and with reduced development timelines [17].

Cancer cells exhibit prominent metabolic reprogramming characterized by a preference for aerobic glycolysis over oxidative phosphorylation, even under sufficient oxygen conditions, a phenomenon known as the Warburg effect [18,19]. This phenomenon is characterized by the conversion of glucose to pyruvate followed by its reduction to lactate, despite a fully functional mitochondrial respiratory chain and complete oxidative capacity for ATP generation in cancer cells. The metabolic shift enables tumor cells to rapidly generate ATP and biosynthetic precursors required for uncontrolled proliferation [20,21]. A key component of this altered metabolism is the upregulation of glucose transporters (GLUTs), which accelerates glucose uptake to sustain elevated glycolysis. Enhanced GLUT expression contributes to the cancer cells’ survival under hypoxic and nutrient-deprived conditions within the tumor microenvironment, promoting invasion and metastasis [22,23]. The Warburg effect has been widely documented across several malignancies, including breast, ovarian, and cervical cancers [24,25,26].

Accumulating data indicate that type 2 diabetes mellitus (T2DM) is associated with an elevated risk of malignancy [27,28]. Metabolic disturbances characteristic of T2DM, including hyperinsulinemia, hyperglycemia, insulin resistance, and chronic inflammation, have been implicated in promoting cancer development [29]. Recent investigations have highlighted the potential of antihyperglycemic agents as promising pharmacological candidates for cancer therapy [30,31]. Among these pharmacological agents, dipeptidyl peptidase 4 (DPP4) inhibitors, commonly used antidiabetic agents in the management of T2DM, have attracted attention as potential therapeutic candidates [32]. This review aims to comprehensively examine the emerging evidence on the repurposing of DPP4 inhibitors for cancer therapy. By integrating insights from epidemiological studies, preclinical investigations, and clinical data, we aim to demonstrate the potential therapeutic benefits and limitations of these antidiabetic agents in cancer management, with a particular focus on their applicability to female-specific cancers.

## 2. Literature Search Strategy

A structured literature search was performed to identify studies investigating the role of DPP4 inhibitors in breast and common gynecological cancers. Electronic bibliographic databases, including PubMed, Web of Science, MEDLINE, and Scopus as well as Google Scholar, were systematically searched for articles published up to April 2026. In the search, the following keywords were used: “DPP4 inhibitor”, “DPPIV inhibitor”, “Dipeptidyl Peptidase-4 inhibitor”, “CD26”, “gliptin”, “sitagliptin”, “saxagliptin”, “linagliptin”, “alogliptin”, “vildagliptin”, “teneligliptin”, “breast cancer”, “breast neoplasm”, “ovarian cancer”, “ovarian neoplasm”, “endometrial cancer”, “endometrial carcinoma”, “cervical cancer”, “cervical neoplasm”, and “gynecologic cancer”.

Studies were considered eligible if they were original research articles that evaluated the impact of DPP4 inhibitors on cancer-related outcomes, including experimental, preclinical, or clinical investigations. Relevant studies examining cancer risk, tumor progression, metastasis, or therapeutic responses were included. Only articles published in English were considered.

Publications such as book chapters, reviews, letters, personal comments, conference abstracts, and articles without full-text availability were excluded. Studies that did not specifically focus on DPP4 inhibition in women with cancer were also omitted.

**Figure 1:**
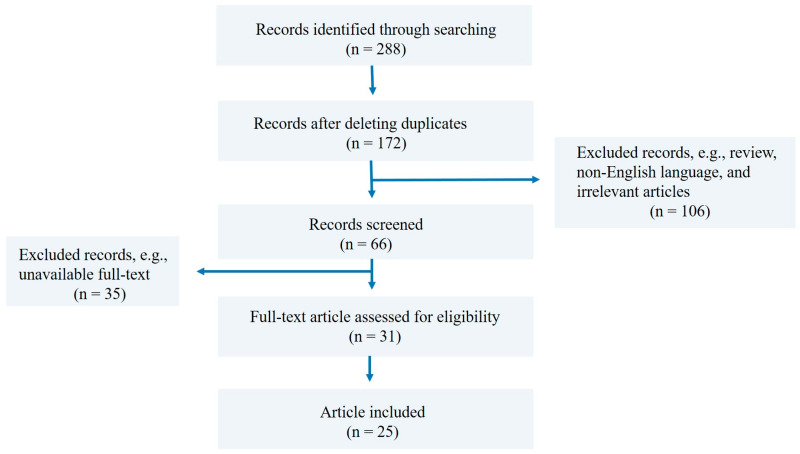
Flow chart of literature search strategy and study selection process.

All retrieved articles were initially screened by title and abstract. Articles that fit the inclusion criteria were selected, and the duplicates were removed, followed by full-text review for eligibility (Figure 1). Additional relevant studies were identified by manually checking the references of included articles.

## 3. Antidiabetic Drug Repurposing in Cancer

Antidiabetic drugs can be classified into several major pharmacological classes based on their mechanisms of action [33,34]. These include insulin or insulin analogs, biguanides, sulfonylureas, thiazolidinediones, sodium–glucose cotransporter 2 (SGLT2) inhibitors, alpha-glucosidase inhibitors, incretin-dependent therapies (GLP1 receptor agonists and DPP4 inhibitors), and meglitinides. Each class lowers blood glucose via distinct physiological pathways (Table 2).

Repurposing existing antidiabetic drugs for cancer therapy has gained significant interest in recent years. Several antidiabetic agents have demonstrated anticancer potential through mechanisms beyond glycemic control, including inhibition of cellular proliferation, induction of apoptosis, and modulation of metabolic and inflammatory pathways. Drug repurposing offers the advantage of utilizing well-characterized drugs with known safety profiles, potentially accelerating the development of effective cancer treatments.

Metformin, a biguanide-class and insulin sensitizer, is widely recognized as the first-line pharmacological treatment for T2DM [35]. It stands out as the most extensively investigated antidiabetic drug for repurposing in cancer treatment. A growing body of evidence from epidemiological studies consistently links metformin use with a reduced risk of overall cancer incidence and cancer-related mortality [36,37,38]. Notably, this effect extends to several site-specific malignancies, including breast, colorectal, liver, pancreatic, endometrial, head and neck, and lung cancers [39,40,41,42,43,44,45], highlighting metformin’s broad potential as an adjunctive agent in oncology. Sulfonylureas are extensively used in low- and middle-income countries for the treatment of T2DM. The influence of sulfonylureas on cancer is unclear. They might exert their anticancer effect by regulating the ATP-binding cassette protein superfamily and ATP-sensitive potassium channels; however, elevated insulin levels can promote tumor growth [46,47]. Various studies have shown potential anticancer effects of sulfonylureas, including induction of apoptosis, decreased inflammation-related lung tumorigenesis, and reduced cell migration and invasion [46,47,48]. In addition, emerging evidence suggests that sulfonylureas may improve the efficacy of certain anticancer agents. A preclinical study demonstrated that a combination of sulfonylureas with doxorubicin exerts synergistic anticancer effects in vitro and in vivo [49]. These findings highlight the complex and context-dependent role of sulfonylureas in cancer therapy. Thiazolidines, also called glitazones, are synthetic compounds that act as agonists of the PPARγ (peroxisome proliferator-activated receptor gamma) receptor. They exhibit antitumor effects that extend beyond their interaction with PPARγ, involving upregulation of PTEN and AMPK, downregulation of Akt/mTOR signaling, and degradation of cyclins D1 and D3 [50]. They also suppress the expression of genes such as the prostaglandin E2 receptor, insulin receptor, and VEGF [51]. Numerous studies have demonstrated that glitazones may exhibit anticancer properties independently of PPARγ stimulation [52,53]. Ongoing studies are exploring the effectiveness of thiazolidine derivatives in treating various cancer types [54,55]. Moreover, SGLT2 inhibitors efficiently reduce glucose levels independent of insulin, making them potential candidates for repositioning in antitumor therapies. Although the exact anticancer mechanism of SGL2 inhibitors has not been fully investigated, many preclinical studies have reported mechanisms that include inhibiting glucose transport, disrupting mitochondrial membrane potential, downregulating β-catenin, PI3K/Akt/mTOR pathways, inducing apoptosis, and contributing to energy depletion, which is essential for cancer progression [56,57]. Accumulating evidence, along with ongoing clinical trials, suggests that combining SGLT2 inhibitors with the established chemotherapeutic regimen may yield therapeutic outcomes [58]. In addition, DPP4 inhibitors are widely used for the treatment of T2DM by prolonging the activities of incretin hormones. Beyond their glucose-lowering activity, DPP4 inhibitors can modulate chemokines and other substrates involved in immune regulation and the tumor microenvironment, suggesting potential therapeutic relevance in cancer [59,60]. Given their multifunctional roles in immune regulation, inflammation, and tumor biology, there is a rationale for focusing on this class in the current review.

## 4. Overview of DPP4 Biology

Dipeptidyl peptidase 4 (DPPIV, CD26) is a type II transmembrane glycoprotein that features a short cytoplasmic region, a 22-amino-acid transmembrane segment, and an extracellular domain [61]. DPP4 influences numerous biological functions through both enzyme-dependent and independent processes, including glucose metabolism, inflammation, immune regulation, cell differentiation, apoptosis, and migration [62,63,64,65]. It has been reported that DPP4 plays a role in the degradation of several peptides, including chemokines, neuropeptides, and incretin hormones such as glucagon-like peptide-1 (GLP-1) and glucose-dependent insulinotropic polypeptide (GIP). Due to DPP4’s cleavage of incretin hormones, DPP4 inhibitors have been used clinically for more than a decade in the management of T2DM [66]. Moreover, the association between DPP4 and adenosine deaminase [67], its interaction with caveolin-1 [68], and its relationship with the extracellular matrix [69] have also been reported. Additionally, DPP4 exerts cell-surface co-receptor activity, mediating viral entry [70]. Furthermore, DPP4 is a key component of the immune response [71]. It is expressed on T lymphocytes and macrophages and modulates responses of B lymphocytes and natural killer (NK) cells [72,73].

### 4.1. DPP4 Expression and Its Role in Cancer

Understanding the biological roles and expression patterns of DPP4 across different tumor types is essential for evaluating its therapeutic potential in cancer. DPP4 is expressed on various cells in solid organs and on most hematopoietic cells. In addition to its expression on the surface of tumor cells, DPP4 is also present in the serum, where its levels may serve as a biomarker of tumor biology in specific tumors and cancer progression [74,75]. DPP4 expression in malignant cells can inhibit tumor progression by promoting cellular differentiation. However, it also modulates local invasion and metastasis. A possible explanation for these conflicting findings may be the diverse and pleiotropic effects of the biologically active DPP4 substrates [76]. Consequently, its impact on cancer must be evaluated individually for each specific tumor type. Accordingly, the following sections summarize current evidence regarding the expression, biological roles, and clinical relevance across different cancer types.

#### 4.1.1. Breast Cancer

##### Samples and Data Analyzed

DPP4 was reported to be expressed heterogeneously in breast cancer, with diverse expression among patient tumor samples, breast cancer cell lines, and experimental animal models [77,78]. In vitro, the expression of CD26 in human breast cancer cell suspensions from different histological subtypes and reference breast cancer cell lines was evaluated by flow cytometry, revealing heterogeneous CD26 expression across patient samples and the cell lines investigated [77]. However, confirmation from other studies with larger sample sizes is required given the limited sample size.

##### Biological and Functional Role

Considerable evidence highlighted the critical role of DPP4 in breast cancer progression, including regulation of cell proliferation, invasion, angiogenesis, metastasis, and chemoresistance. As an example, an in vitro study reported that TNF-α elevates DPP4 expression, thereby activating the MAPK/ERK signaling pathway [79]. Consistently, another study identified a potential mechanism by which DPP4 promotes epithelial–mesenchymal transition (EMT) in breast cancer cells in vitro, augmenting PIN1 expression by activating the transcription factor E2F1 in response to epidermal growth factor (EGF) [80].

DPP4 has also been implicated in metastatic dissemination. Its expression on the surface of lung capillary endothelial cells in rats was found to serve as a receptor for rat breast cancer cells that display fibronectin and promote lung metastasis. Additionally, the extent of binding was revealed to be proportional to the amount of fibronectin in the breast cancer cells [81]. Furthermore, peptides comprising the fibronectin DPP4-binding domain blocked the DPP4-fibronectin interaction and reduced pulmonary metastasis of the breast cancer cells [82]. Donnenberg et al. described surface markers of metastatic breast cancer cells obtained from breast cancer cell lines. This model consists of human mammary epithelial cells, HMLER cells, tumorigenic at low frequency, and HMLER cells transduced with Twist (transcription factor) that are invasive and metastatic. The study highlighted a specific upregulation of DPP4 expression [78].

##### Role in the Tumor Microenvironment

Within the tumor microenvironment (TME), DPP4 expression has been associated with specific fibroblast populations. In mouse models of breast cancer, normal fibroblasts can differentiate into distinct cancer-associated fibroblast (CAF) subsets based on DPP4 expression. In particular, DPP4+ normal fibroblasts evolve into protumorigenic inflammatory CAFs, promoting tumor cell invasion, recruiting monocytes in a CXCL12-dependent manner, and increasing matrix metalloproteinase (MMP) activity [83]. Human breast stromal fibroblasts were distinguished according to CD26 and CD105 expression levels into two different fibroblast groups: lobular fibroblasts (CD105high/CD26low) and interlobular fibroblasts (CD105low/CD26high). Among these, the lobular fibroblasts exhibit mesenchymal stem cell properties that promote epithelial cell development and morphogenesis. This might provide the microenvironment for human breast luminal epithelial progenitor cells [84].

##### Clinical Relevance

These findings suggest that DDP4 contributes to tumor progression, metastasis, and microenvironmental regulation in breast cancer, highlighting its potential as a therapeutic target and biomarker for disease progression.

#### 4.1.2. Cervical Cancer

##### Samples and Data Analyzed

DPP4 expression and activity in cervical cancer cell lines have been investigated using cervical cancer cell lines and genomic analyses of tumor samples. A study investigated DPP4 expression and activity in cervical cancer cell lines (HeLa, SiHa, and C33A) and the non-tumorigenic HaCaT cell line. Another study determined genes associated with cervical cancer development by screening for those that overlapped between expressed and methylated genes in cervical cancer samples [85].

##### Biological and Functional Role

These studies demonstrated variable DPP4 expression among cervical cancer cell lines. DPP4 expression was detected in SiHa, C33A, and HaCaT cell lines. Whereas HeLa cells demonstrated almost undetectable expression levels. The study also reported a higher migratory ability of HeLa, compared to SiHa. Demonstrating the role of DPP4 in regulating cell migration. Moreover, DPP4 inhibition reduced the adhesion capacity of SiHa and HeLa cells. However, the authors indicated that cell adhesion regulation was independent of DPP4 [86]. In addition, genomic analysis identified DPP4 alongside four other genes as simultaneously downregulated and hypermethylated in cervical cancer samples, indicating its potential involvement in cervical cancer pathogenesis, receptor binding, and ameboid-type cell migration [85].

##### Clinical Relevance

Collectively, studies suggest that DPP4 plays a role in cervical cancer biology, promoting processes such as cell migration, adhesion, and gene expression regulation; however, further research is needed to understand its role in cervical cancer development and its potential value as a therapeutic target and biomarker.

#### 4.1.3. Ovarian Cancer

##### Samples and Data Analyzed

The role of DPP4 in ovarian cancer has been investigated using ovarian tumor tissues, patient serum samples, ovarian cancer cell lines, and animal models. Studies have examined DPP4 expression at both the mRNA and protein levels, as well as its functional role in tumor progression and metastasis [87,88,89,90,91].

##### Biological and Functional Role

DPP4 has been implicated in several aspects of ovarian cancer biology, including tumor invasion, metastasis, and regulation of the tumor microenvironment. Under hypoxic conditions, ovarian cancer cells upregulated DPP4 mRNA expression. However, the specific activity of secreted DPP4 decreased due to inactivation and proteolytic shedding mediated by matrix metalloproteinases, particularly MMP10 and MMP13. This suggests a complex regulation of DPP4 in the tumor microenvironment [88]. Ovarian cell lines, including HRA, SKOV3, TAOV, NOS4, and NOS2, were compared regarding their DPP4 expression and invasive potential. HRA and SKOV3 expressed a low level of DPP4 but showed the highest invasive potential. Meanwhile, other cell lines expressed a high level of DPP4 but exhibited low invasive potential [89]. Transfection of SKOV3 cells with DPP4 altered cellular morphology and increased adhesion; however, migration and invasion were substantially reduced. Moreover, nude mice inoculated with DPP4-transfected SKOV3 cells exhibited substantially less peritoneal dissemination and lived approximately twice as long as those receiving the parental or vector-transfected cells. TGFβ1 and miR29a-3p were found to downregulate DPP4 expression, leading to reduced proliferation and invasion of ovarian cancer cells [90]. DPP4 expression was upregulated in rats with polycystic ovary syndrome (PCOS). Silencing DPP4 expression induced ovarian granulosa cell proliferation and activated the cAMP response element-binding protein (CREB)/aromatase pathway [91].

##### Clinical Relevance

DPP4 expression was connected with lymph node metastasis and the clinical stage of the disease. However, no correlation was found between tumor type, histological grade, or disease-free survival. Further, the study revealed that 97.67% of malignant epithelial ovarian cancer samples were positive for DPP4 mRNA, suggesting the prognostic value of DPP4 in ovarian cancer [87]. In contrast, in another study, the serum DPP4 level in patients with ovarian tumors is reduced, and the decrease is more pronounced in patients with poor prognosis [92]. Another study reported that DPP4 mRNA and protein levels were reduced in ovarian tumor tissues. Additionally, patients with ovarian cancer had lower serum DPP4 levels compared to healthy subjects. Kaplan–Meier plot analysis revealed that higher DPP4 expression was associated with worse prognosis in these patients [90]. These conflicting findings warranted a further evaluation of DPP4’s role as a prognostic biomarker in ovarian cancer.

#### 4.1.4. Endometrial Cancer

##### Samples and Data Analyzed

The role of DPP4 in endometrial cancer has been investigated using endometrial tumor tissues, endometrial carcinoma cell lines, and experimental in vitro and in vivo models. Studies have evaluated both the expression levels of DPP4 and its involvement in signaling pathways associated with tumor progression [93,94,95].

##### Biological and Functional Role

DPP4 was expressed in normal endometrial glandular cells, but its expression in endometrial adenocarcinoma decreased with increasing tumor grade. Regulated on activation, normal T cell expressed and secreted (RANTES), one of the substrates of DPP4, was found to be overexpressed in endometrial carcinoma, promoting the proliferation of endometrial adenocarcinoma cell lines. The study suggested a beneficial effect of DPP4 inhibition on endometrial adenocarcinoma progression, due to the loss of degrading activity of bioactive factors such as RANTES [93]. In contrast, another study examined the relationship between DPP4 expression and tumor progression in endometrial carcinoma and found that increased DPP4 levels altered cell morphology and enhanced proliferation, invasion, and carcinogenesis, both in vitro and in vivo. Overexpression of DPP4 also elevated hypoxia-inducible factor 1α (HIF-1α) and vascular endothelial growth factor A (VEGFA), thereby activating the HIF-1α-VEGFA signaling pathway [94]. Further supporting the relevance of DPP4 inhibition in endometrial cancer therapy, a recent study examined the functions and signaling pathways regulated by DPP4 in endometrial carcinoma. The study reported that DPP4 is essential for endometrial carcinoma cell proliferation in vitro and in vivo tumorigenesis through the interleukin-6 (IL-6)/signal transducer and activator of transcription 3 (STAT3) pathway. Inhibition of DPP4 suppressed IL-6 secretion and reduced STAT3 expression, thereby decreasing tumor proliferation [95].

##### Clinical Relevance

These findings indicate that DPP4 may contribute to endometrial cancer progression through multiple signaling pathways, although its precise role remains controversial. Some studies suggest a potential therapeutic benefit of DPP4 inhibition, whereas others suggest that DPP4 overexpression may support tumor progression. Therefore, further research is required to clarify the clinical significance of DPP4 as a potential therapeutic target in endometrial cancer.

Collectively, these findings indicate that the role of the DPP4 in cancer remains complex and a matter of controversy. These discrepancies may arise from several biological and methodological factors. DPP4 exists in both membrane-bound and soluble forms, which may exhibit distinct biological effects in tumor progression and immune modulation [96]. In addition, DPP4 exerts both enzymatic and non-enzymatic functions, allowing it to regulate many signaling pathways and participate in protein–protein interactions that influence tumor progression [75]. The effects of DPP4 might also depend on cellular context, including tumor-cell-intrinsic mechanisms as well as stromal or immune-related mechanisms. For example, DPP4 inhibition can enhance antitumor immune responses by modulating chemokine activity [97], while in other contexts it might influence tumor cell migration or EMT [98]. In addition, differences among DPP4 inhibitors, including selectivity, off-target effects, and pharmacokinetics, together with experimental models and drug dosages, were suggested to contribute to the variable outcomes across studies [60]. Nevertheless, accumulating evidence has highlighted the role of DPP4 in the progression of female cancers, including tumor proliferation, invasion, metastasis, and tumor-microenvironment interactions, suggesting that DPP4 inhibitors may represent a promising therapeutic strategy and highlighting the possible diagnostic and prognostic relevance of DPP4 expression.

### 4.2. The Effects of DPP4 in the Immune System

DPP4 plays a role in immunoregulation through both enzymatic and non-enzymatic mechanisms. Firstly, its catalytic activity cleaves multiple chemokines, cytokines, and regulatory peptides, thereby directly affecting their immunological responses. Secondly, DPP4 functions as a costimulatory molecule involved in T-cell receptor signaling and activation [99].

Through non-catalytic protein–protein interactions, DPP4 can bind several molecules, including fibronectin, caveolin-1, adenosine deaminase (ADA), and CXC chemokine receptor 4 (CXCR4), which participate in immune signaling pathways [82,99,100]. One of the best-characterized interactions is the CD26-ADA complex. ADA catalyzes the hydrolytic deamination of adenosine to inosine, and high concentrations of adenosine are known to inhibit the T-lymphocyte proliferation [101,102]. The binding of ADA to CD26 counteracts this inhibitory effect by reducing extracellular adenosine levels, thereby promoting T-cell activation and proliferation. Accordingly, the CD26/ADA/adenosine pathway is considered an important mechanism in T-cell activation and immune responses.

CD26 is highly expressed on activated and proliferating T lymphocytes and is particularly enriched in specific immune cell populations, including mucosal-associated invariant T (MAIT) cells, CD4+ and CD8+ effector memory cells, Th17 cells, and NKT cells. In contrast, naïve CD8+CD45RA+, CD4+CD45RA+, and CD4+CD25+ Treg tend to have lower expression levels [103,104]. Functional studies have shown that CD26 can interact with caveolin-1 on antigen-presenting cells, thereby promoting the upregulation of costimulatory molecules, such as CD86, and enhancing T-cell activation [68]. In addition, crosslinking of CD26 can stimulate T-cell proliferation and signaling [105]. In addition to its role in lymphocyte activation, CD26 may also contribute to immune cell migration and adhesion. For example, CD4+ T cells, which induce in vitro transendothelial migration, appear to highly express CD26, suggesting a possible role for CD26 in T cell migration [71].

Furthermore, the enzymatic activity of DPP4 may regulate inflammatory responses by cleaving and inactivating chemokines, thereby shaping the chemokine gradients and limiting immune cell recruitment once inflammatory cells have accumulated at the target site [106]. However, the immunological role of DPP4 remains somewhat controversial. These differences reflect the high context-dependent nature of DPP4 function and the differences in expression patterns across species. Notably, significant differences exist between the human and murine immune systems, including variation in CD26 expression across immune cell populations and the unique ability of human CD26 to bind ADA. These differences should be carefully considered when extrapolating from experimental animal models [106,107].

## 5. DPP4 Inhibition in Cancer Treatment

Due to elevated DPP4 levels in the tumor microenvironment and its suggested role in promoting tumor growth and progression in specific cancer types, multiple studies have explored the anticancer properties of DPP4 inhibitors.

### 5.1. Breast Cancer

In breast cancer, the potential antitumor effects of sitagliptin have also been examined. A study demonstrated a positive correlation between DPP4 expression and PIN1, a key regulator of phosphorylation signaling involved in cell proliferation and transformation in human breast cancer tissues. The study findings showed that sitagliptin reduced epithelial transformation by downregulating PIN1 expression, suggesting that DPP4 may function upstream in the PIN1 signaling pathway [80]. A study investigated the effects of sitagliptin and vildagliptin on the triple-negative breast cancer (TNBC) cells MDA-MB-231. 72 h of treatment with gliptins significantly decreased cell viability, migration, and invasion. The study also showed that both drugs induced apoptosis, reduced lactate production, and increased the mitochondrial DNA-to-nuclear DNA ratio. Upregulation of transcription factors such as PGC-1α, NRF-1, NRF-2, TFAM, and HO-1 was also detected [108]. Notably, pretreatment with sitagliptin or vildagliptin increased TNBC cells’ sensitivity to doxorubicin, a commonly used chemotherapeutic agent. These findings suggest that gliptins may exert anticancer effects in TNBC through metabolic reprogramming and could serve as adjuvants to chemotherapy [108]. In contrast, another study reported that the DPP4 inhibitor linagliptin did not affect breast cancer cell proliferation in vitro [109]. A study investigated the effects of the DPP4 inhibitors saxagliptin and sitagliptin on murine breast cancer cells (4T1 cell line). The findings indicated that DPP4 inhibitors significantly enhanced breast cancer metastatic capabilities in vitro and in vivo. The study suggested that the pro-metastatic effects were mediated through the reactive oxygen species (ROS)-NRF2-HO-1 axis, highlighting a potential oncogenic pathway activated by DPP4 inhibition [110]. In addition, it was reported that inhibiting DPP4 in 4T1 breast cancer cells, either by treatment with the DPP4 inhibitor KR62436 (KR) or by DPP4 knockdown, led to breast cancer metastasis via upregulation of CXCL12/CXCR4, which activates mTOR to promote EMT. Whereas treatment with the CXCR4 inhibitor AMD3100 or the mTOR inhibitor rapamycin blocked KR-induced EMT in cancer cells, and AMD3100 suppressed KR-induced metastasis in vivo [98]. Another study revealed that inhibiting DPP4 induces autophagy via the CXCL12/CXCR4/mTOR/HIF-1α pathway, thereby enhancing breast cancer cell survival. Interestingly, metformin reversed this effect by promoting apoptosis in breast cancer cells, thereby reducing autophagy [111]. Further studies have shown that DPP4 inhibition correlates with activation of the mTOR signaling pathway [112,113]. On the other hand, a study that investigated the effects of the DPP4 gene family on breast cancer prognosis using publicly available databases showed that high expression levels of DPP4 were correlated with poor survival in breast cancer patients [114]. Meanwhile, a cohort study investigating the impact of DPP4 inhibitors among patients with diabetes and newly diagnosed breast cancer found no increased risk of cancer metastases (adjusted hazard ratio (aHR), 95% confidence interval (CI): 1.00, 0.49–2.02) [115]. A meta-analysis examined the association between the use of gliptins and breast cancer development and reported no significant association compared with placebo or other active drugs (Risk ratio, 95% CI: 0.72, 0.50–1.06, *p* = 0.094) [116]. In contrast, a study evaluating the survival outcomes of DPP4 inhibitors in breast cancer patients found a non-favorable effect among DPP4 inhibitor users compared with the non-user control group (HR, 95% CI: 1.07, 0.93–1.25, *p* = 0.33). However, the study reported significantly improved patient survival among users of metformin alone or in combination with DPP4 inhibitors, with HRs of 0.79 (95% CI: 0.74–0.84, *p* < 0.001) and 0.73 (95% CI: 0.62–0.85, *p* < 0.001), respectively [117]. A meta-analysis by Noh et al. found that DPP4 inhibitors did not significantly increase the patient’s risk of cancer metastasis evaluated in different primary cancer types, including breast cancer, when compared to diabetic cancer patients with no antidiabetic treatment [118].

### 5.2. Cervical Cancer

In a cervical cancer study, the inhibitory effects of linagliptin on HeLa cell proliferation were evaluated. Linagliptin demonstrated a time-dependent cytotoxic effect, inhibiting cell proliferation at 24 and 72 h. Molecular docking studies revealed that linagliptin binds effectively to human heat shock protein 90 (Hsp90), suggesting a potential mechanism for its anticancer activity [119]. Another study investigated the role of DPP4 in the migration of cervical carcinoma cells. The study found that the differential expression and enzymatic activity of DPP4 significantly affect the migration ability of cervical carcinoma cells, suggesting that DPP4 inhibitors could modulate cancer cell migration. In addition, in the presence of sitagliptin phosphate, SiHa and HeLa cervical cells exhibited a significant reduction in adhesion [86].

### 5.3. Ovarian Cancer

In ovarian cancer, Kosowska et al. (2020) found that sitagliptin enhances the response of ovarian cancer cells to the chemotherapeutic agent paclitaxel by promoting apoptosis through caspase 3/7 activation, maintaining paclitaxel’s effects on ERK and Akt signaling, reducing cell migration and invasiveness, and decreasing levels of MMPs and tissue inhibitors of metalloproteinases (TIMPs) in SKOV-3 cells treated with sitagliptin and paclitaxel. These results suggest that sitagliptin may enhance the efficacy of chemotherapy in ovarian cancer [120]. Moreover, a study demonstrated that sitagliptin enhances immune responses in a syngeneic ovarian cancer mouse model by increasing CXCR3-mediated CD8+ T-cell infiltration, reducing immunosuppressive cytokines and Treg recruitment, leading to decreased metastatic burden and prolonged survival. However, combining sitagliptin with paclitaxel negated these immune benefits, suggesting sitagliptin may be more effective as an adjunct therapy between chemotherapy cycles [121].

### 5.4. Endometrial Cancer

In endometrial carcinoma, a study demonstrated that DPP4 overexpression in endometrial carcinoma cells altered cell morphology and increased proliferation, invasion, and tumorigenesis, both in vitro and in vivo. These effects were mediated through the activation of the HIF-1α/VEGFA signaling pathway. Importantly, DPP4 inhibition by sitagliptin suppressed these tumor-promoting effects, suggesting that sitagliptin may be an effective therapeutic agent against endometrial carcinoma [94]. A study evaluated the risk reduction in endometrial cancer associated with DPP4 inhibitors in women with type 2 diabetes, comparing their use with sulfonylureas. The study concluded that their use was not associated with a decreased risk of endometrial cancer [122].

### 5.5. Cross-Cancer Mechanistic Insight of DPP4 Inhibition

**Figure 2:**
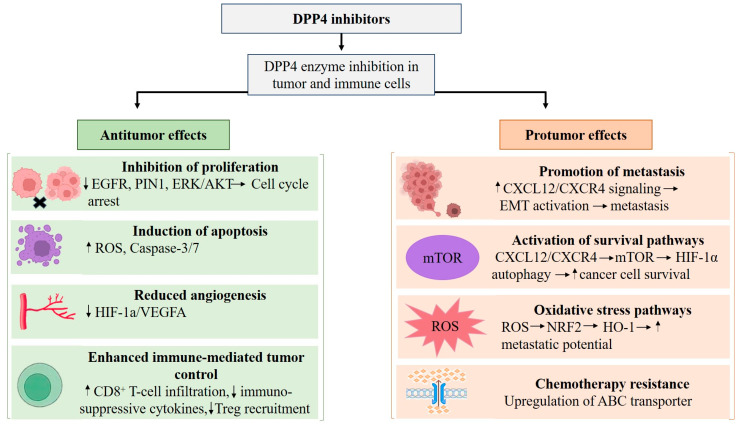
Protumor and antitumor effects of DPP4 inhibition in female cancers. Note: ↑ indicates an increase; ↓ indicates a decrease.

When examining the available evidence across different female malignancies, several common mechanistic patterns emerge regarding the role of DPP4 and its pharmacological inhibition. In breast cancer, studies suggest a dual role in which DPP4 inhibition may exert anticancer effects through metabolic reprogramming and enhanced chemotherapy sensitivity, yet may also promote metastatic potential through activation of signaling pathways such as CXCL12/CXCR4/mTOR/EMT axis and ROS-NRF2-HO-1 signaling [98,110,112,113]. In contrast, in endometrial cancer, DPP4 overexpression promotes tumor progression via activation of the HIF-1α/VEGFA pathway, while pharmacological inhibition of tumor growth and invasion [94]. In the ovarian cancer model, DPP4 inhibition has been associated with improved chemotherapy responses and enhanced antitumor immune activity, through increased CD8+ T-cell infiltration and modulation of the tumor immune microenvironment [121]. Meanwhile, in cervical cancer, available evidence suggests that DPP4 activity may be involved in cancer cell proliferation and migration [86,119], in which cells exhibited a significant reduction in adhesion when sitagliptin phosphate was present, although the underlying mechanisms remain less well defined. Collectively, these observations indicate that the biological impact of DPP4 and its inhibition is context-dependent and may vary by tumor type, dominant signaling pathways, and microenvironmental interactions. Molecular pathways implicated across malignancies include ERK signaling, CXCL12/CXCR4-mediated migration and metastasis, HIF-1α/VEGFA-driven angiogenesis, and immune microenvironment remodeling (Figure 2).

### 5.6. Translational Considerations for Repurposing DPP4 Inhibitors

Translational considerations are particularly important when evaluating the potential repurposing of DPP4 inhibitors in oncology. It is essential to acknowledge that many studies examining the anticancer potential of DPP4 inhibitors employ concentrations significantly higher than those used in clinical practice for diabetes management. While gliptins inhibit DPP4 effectively at nanomolar levels in antidiabetic settings (typically achieving plasma concentrations below 2 µM in humans), anticancer research often employs much higher doses [60,123]. In some cases, these concentrations exceed what can be realistically achieved in vivo. These discrepancies raise important questions regarding whether the observed antitumor effects reflect true on-target inhibition or rather off-target pharmacological actions occurring at supratherapeutic concentrations. Furthermore, variability in pharmacokinetic properties among different gliptins, including differences in bioavailability, tissue distribution, and enzyme selectivity, may further influence their potential anticancer activity. Taken together, these considerations highlight a key limitation in translating preclinical findings into clinical oncology and underscore the need for further studies to carefully evaluate clinically achievable drug exposures when assessing the feasibility of repurposing DPP4 inhibitors for cancer therapy. Altogether, the findings (summarized in Table 3) are controversial and scarce regarding the potential anticancer effects of DPP4 inhibitors and their role in cancer outcomes. Therefore, careful consideration and individualized assessment are necessary when prescribing DPP4 inhibitors to women with cancer.

## 6. Conclusions

DPP4 inhibition is a standard therapeutic modality in patients with T2DM. Gliptins are orally administered DPP4 inhibitors that are widely prescribed due to their favorable safety profile. Although current evidence remains inconclusive, findings from the studies reviewed here indicate that gliptins may offer potential advantages in certain contexts for patients who have cancer or are at increased risk of developing malignancies. However, the molecular mechanisms responsible for their suggested anticancer effects remain largely undefined. Growing insight into the role of DPP4 enzymatic activity in regulating incretins and other biopeptides suggests that gliptins could be considered for therapeutic repurposing in oncology.

Current data indicate that DPP4 inhibition may be promising in settings where it enhances chemotherapy sensitivity or modulates the tumor immune microenvironment, as suggested by studies in ovarian cancer models and certain breast cancer studies. Conversely, caution may be warranted in tumor contexts where DPP4 inhibition has been associated with activation of metastasis-related pathways, such as the CXCL12/CXCR4/mTOR/EMT axis and ROS-NRF2-HO-1 signaling observed in some breast cancer models. While the modulation of DPP4 activity is unlikely to be a critical component of anticancer therapies, it may provide benefits in selected tumor types or under selected conditions. The determination of these specific circumstances remains an objective for future preclinical and clinical investigations.

## Figures and Tables

**Table 1:** Overview of major female cancers.

Cancer Type	Worldwide Incidence in 2022	Worldwide Mortality in 2022	Stage/Clinical Setting	First-Choice Systemic Anticancer Drug(s)/Regimen
Breast Cancer	2,292,686	665,684	Early-stage HR+/HER2−	Tamoxifen/Aromatase inhibitors
			Early-stage HER2+	Trastuzumab + Paclitaxel/Docetaxel
			Triple-negative (TNBC), Stage II–III	Anthracycline + Cyclophosphamide followed by paclitaxel
			Locally advanced	Anthracycline-based regimen ± HT ± Trastuzumab
			Metastatic HR+	CDK4/6 inhibitor + Aromatase inhibitor
			Metastatic HER2+	Trastuzumab + Pertuzumab + Taxane
Ovarian Cancer	324,398	206,839	Stage I (high-risk)/II–IV	Carboplatin + Paclitaxel
			Advanced Stage III–IV	Carboplatin + Paclitaxel + Bevacizumab
			BRCA-mutated maintenance	Olaparib/Niraparib
			Platinum-resistant recurrence	Pegylated liposomal doxorubicin/Weekly paclitaxel/Bevacizumab
Cervical Cancer	661,021	348,189	Locally advanced (IIB–IVA)	Weekly cisplatin with radiotherapy
			Metastatic/Recurrent	Paclitaxel + Cisplatin/Carboplatin + Bevacizumab
			PD-L1-positive metastatic	Pembrolizumab + Platinum-based chemotherapy
Endometrial Cancer	420,242	97,704	Stage I–II low risk	Usually no systemic therapy
			High-risk/Stage III–IV	Carboplatin + Paclitaxel
			Hormone receptor-positive recurrent/low-grade	Progestins/Aromatase inhibitors
			Advanced/recurrent MSI-H/MMRd	Pembrolizumab/Dostarlimab
			Advanced/recurrent MMR-proficient	Pembrolizumab + Lenvatinib

Data summarized from references [4,9,10,11,12,13,14,15,16].

**Table 2:** Major classes of antidiabetic drugs.

Drug Class	Representative Drug(s)	Year/Period of FDA Approval	Primary Antidiabetic Mechanism
Insulin and insulin analogs	Regular insulin, insulin glargine, insulin aspart, insulin detemir, and insulin degludec	1982–2015	Directly lowers blood glucose by promoting cellular glucose uptake and inhibiting hepatic glucose production
Biguanide	Metformin	1995	Reduction in hepatic gluconeogenesis, improved insulin sensitivity
Alpha-glucosidase inhibitors	Acarbose and miglitol	1995–1996	Modifying the intestinal digestion of carbohydrates
Sulfonylureas	Glyburide, glimepiride, and glipizide	1984–1995	Stimulation of insulin secretion
Thiazolidinediones	Pioglitazone and rosiglitazone	1999	Activation of PPARγ leads to improved insulin sensitivity
Sodium-glucose cotransporter 2 inhibitors	Dapagliflozin, empagliflozin, and canagliflozin	2013–2014	Block renal glucose reabsorption
Meglitinides	Nateglinide and repaglinide	1997–2000	Stimulation of insulin secretion
GLP1 receptor agonists	Exenatide, liraglutide, and semaglutide	2005–2017	Inhibition of pancreatic glucagon secretion and reduction in gastric emptying
DPP4 inhibitors	Sitagliptin, saxagliptin, linagliptin, and alogliptin	2006–2013	DPP4 inhibition prevents the degradation of incretin (GLP-1 and GIP)

Abbreviations: PPARγ, peroxisome proliferator-activated receptor gamma; GLP-1, glucagon-like peptide-1; GIP, glucose-dependent insulinotropic polypeptide.

**Table 3:** Descriptive overview of studies on DPP4 anticancer effects in female cancers.

Cancer Type	DDP4 Inhibitor	Study Model/Sample Type	Key Mechanisms	Clinical Relevance	Reference
Breast cancer	Sitagliptin	Human breast cancer tissues	Downregulation of PIN1 expression	DPP4 may function upstream of PIN1	[80]
	Sitagliptin and vildagliptin	TNBC cells (MDA-MB-231)	Upregulation of PGC-1α, NRF-1, NRF-2, TFAM, HO-1 and mitochondrial regulation	Treatment with gliptins increased sensitivity to doxorubicin	[108]
	Saxagliptin and sitagliptin	Murine breast cancer cells (4T1 cell line)	ROS-NRF2-HO-1 axis	Potential protumor effects of DPP4 inhibition leading to increased metastasis	[110]
	Sitagliptin	MCF-7 cells	Down-regulation of KAT7 and SIRT1 gene expression	Sitagliptin has effects on the histone epigenetic landscape	[124]
	DPP4 inhibitors	4T1 cells and an allograft mouse model	CXCL12/CXCR4/mTOR/TGFβ and ABC transporters	DPP4 inhibitors potentiate chemotherapy resistance	[125]
	Sitagliptin	Preclinical mouse and tumor cells model	Increases chemokine CCL11 level and increases the migration of eosinophils and IL-33	DPP4 inhibition leading to IL-33- and eosinophil-mediated antitumor immune response	[97]
	Sitagliptin	Ehrlich tumor—bearing mice	Modulation of MDA and GSH, IL-6 and IL-1β, and VEGF, reducing β-catenin and cyclin-D1 and enhancement of survivin, p53, caspase 3	Sitagliptin reduces tumor growth, and its chemosensitizing doxorubicin anticancer activity	[126]
	Teneligliptin	MCF-7 cells	No mechanistic insight	Dose-dependent reduction in cell viability	[127]
	Sitagliptin, vildagliptin, saxagliptin, alone or in combination with other antidiabetic agents	Cohort study	–	No increased risk of breast cancer	[128]
	Sitagliptin, vildagliptin, and saxagliptin	Cohort study	–	The use of DPP4 inhibitors was not linked to an increased risk of metastasis	[115]
	Alogliptin, linagliptin, saxagliptin, sitagliptin, and vildagliptin	Cohort study	–	DPP4 inhibitor use has been associated with improved survival in patients with prostate cancer, but not in those with breast or pancreatic cancer	[117]
	Not specified	Meta-analysis	–	DPP4 inhibitors did not significantly increase the patient’s risk of breast cancer metastasis	[118]
	Sitagliptin	Cohort study	–	Epidemiological association suggesting sitagliptin may reduce breast cancer risk	[129]
Cervical cancer	Sitagliptin	SiHa and HeLa cells	DPP4 enzymatic activity	Sitagliptin phosphate led to a marked decrease in adhesion in both SiHa and HeLa cell lines	[86]
	Linagliptin	HeLa cells	Interaction with human heat shock protein 90 (Hsp90)	Reduction in cell proliferations	[119]
Ovarian cancer	Sitagliptin	SKOV-3 cells	Caspase 3/7 activation, ERK/Akt signaling, and suppressing MMPs	Chemotherapy sensitization	[120]
	Sitagliptin	In vitro and in vivo mouse model	Increasing CXCR3-driven CD8+ T-cell infiltration	Immunomediated antitumor effects and prolonging survival	[121]
Endometrial cancer	Sitagliptin	In vitro and in vivo	HIF-1α/VEGFA signaling cascade inhibition	Suppressing tumor-promoting effects	[94]
	Alogliptin, linagliptin, saxagliptin, sitagliptin, and vildagliptin	Cohort study	–	No reduced endometrial cancer risk	[122]

Abbreviations: EGF, epidermal growth factor; ROS, Reactive oxygen species; GLP-1, glucagon-like peptide-1; HIF, hypoxia-inducible factor; VEGFA, vascular endothelial growth factor A; MDA, malondialdehyde; GSH, glutathione.

## Data Availability

No new data were created or analyzed in this study. Data sharing is not applicable to this article.

## Abbreviations

The following abbreviations are used in this manuscript:

CAFs	Cancer-associated fibroblasts
DPP4	Dipeptidyl peptidase 4
EGF	Epidermal growth factor
EMT	Epithelial–mesenchymal transition
GIP	Glucose-dependent insulinotropic polypeptide
GLP-1	Glucagon-like peptide-1
GLUT	Glucose transporter
HIF	Hypoxia-inducible factor
Hsp90	Human heat shock protein 90
IL-6	Interleukin-6
MMP	Matrix-metalloproteinase
NFkB	Nuclear factor kappa B
PCOS	Polycystic ovary syndrome
PPARγ	Peroxisome proliferator-activated receptor gamma
ROS	Reactive oxygen species
STAT3	Signal transducer and activator of transcription 3
T2DM	Type 2 diabetes mellitus
TNBC	Triple-negative breast cancer
VEGF	Vascular endothelial growth factor
